# ATM controls DNA repair and mitochondria transfer between neighboring cells

**DOI:** 10.1186/s12964-019-0472-x

**Published:** 2019-11-08

**Authors:** Sha Jin, Nils Cordes

**Affiliations:** 1 OncoRay – National Center for Radiation Research in Oncology, Faculty of Medicine, Technische Universität Dresden, D–01307 Dresden, Germany; 2 Department of Radiation Oncology, University Hospital Carl Gustav Carus, Technische Universität Dresden, D–01307 Dresden, Germany; 3 Helmholtz–Zentrum Dresden – Rossendorf, Institute of Radiooncology – OncoRay, D–01328 Dresden, Germany; 4 German Cancer Consortium (DKTK), partner site Dresden, D–69192 Heidelberg, Germany; 5 German Cancer Research Center (DKFZ), D–69192 Heidelberg, Germany

**Keywords:** Cell–cell communication, Genotoxic stress, Mitochondria exchange, DNA damage repair, Ataxia telangiectasia mutated (ATM)

## Abstract

Intercellular communication is essential for multicellular tissue vitality and homeostasis. We show that healthy cells message protective signals through direct cell–cell connections to adjacent DNA–damaged cells in a microtubule–dependent manner. In DNA–damaged cells, mitochondria restoration is facilitated by fusion with undamaged mitochondria from healthy cells and their DNA damage repair is optimized in presence of healthy cells. Both, mitochondria transfer and intercellular signaling for an enhanced DNA damage response are critically regulated by the activity of the DNA repair protein ataxia telangiectasia mutated (ATM). These healthy–to–damaged prosurvival processes sustain normal tissue integrity and may be exploitable for overcoming resistance to therapy in diseases such as cancer.

## Background

In multicellular organisms, neighboring cells continuously exchange information for coordinating homeostasis, survival and development. Direct communication through cell–cell contact is one of the most succinct ways to exchange information [1] as demonstrated by transfer of small organelles like mitochondria carrying signaling molecules such as RNA [2–4]. Under stress conditions, mitochondria often move from one cell to another and between different cell types [1, 5–7]. Limited mitochondrial functionality results from exposure to DNA–damaging agents like ionizing radiation, which is rescued by mitochondria fission and fusion [8, 9].

A prominent process for intercellular communication is known as bystander effect [10] describing a DNA damage response in healthy cells triggered by signals from damaged cells [11]. Among the various types of DNA damage, DNA double–strand breaks (DSB), as most life–threatening lesions, are repaired through non–homologous end joining (NHEJ) and homologous recombination (HR). Upon DSB sensing [12], ataxia–telangiectasia mutated (ATM) and DNA protein kinase catalytic subunit (DNA–PKcs) phosphorylate numerous substrates including histone H2AX and p53 binding protein 1 (53BP1) [13–15]. A variety of signaling molecules such as ATM [16], nitric oxide (NO) and reactive oxygen species (ROS) have been shown to be relayed through gap junctions between DNA damaged and healthy cells [17]. Remaining is the important and yet open question how healthy cells influence DNA–damaged cells.

Here, we designed a co–culture model system containing human pancreatic cancer cells and ATMwt as well as ATM^−/−^ fibroblasts, in which genotoxically injured cells were co–cultured with healthy cells upon removal of secreted factors putatively connectable to bystander effects. We comparatively characterized the DSB repair in target and non–target cell populations under co– and mono–culture conditions. Our results show that the presence of healthy cells profoundly modifies the DNA damage response of genotoxically injured cells by a microtubule– and ATM–dependent exchange of healthy mitochondria.

## Materials and methods

### Cell culture

Human pancreatic cancer cells (MiaPaCa–2), wild–type (wt) and ATM^−/−^ fibroblasts, MiaPaCa–2–GFP, MiaPaCa–2–G1G2, ATMwt–GFP and ATM^−/−^–GFP were grown in Dulbecco’s Modified Eagle Medium (DMEM, without phenol red, Gibco) containing 10% (v/v) fetal bovine serum, 1% (v/v) non–essential amino acids, 4 mM L–Glutamine, 100 units/ml penicillin–streptomycin, 4.5 g/L D–glucose. Cells were maintained at 37 °C with 5% CO_2_. All cells were tested negative for mycoplasma. MiaPaCa–2 cells were purchased from the American Type Culture Collection (ATCC). Human fibroblasts (ATMwt; ATM^−/−^) were kindly provided by P. A. Jeggo (University of Sussex, UK) [18].

### Plasmid DNA transfection

Cells were plated onto uncoated 35 mm dishes with a 0.17 mm glass bottom (MatTek) and allowed to reach 60–70% confluency. GFP empty vector or Fucci cell cycle indicator series (Amalgaam) were introduced into the cells using Lipofectamine 2000 (Invitrogen,) according to the manufacture’s protocol. Briefly, cells were incubated in 250 μl OptiMEM with a DNA–mix of 250 μl (containing plasmid DNA of 1–2 μg / μl and 10 μl Lipofectamine 2000 in 240 μl OptiMEM). Transfection media was removed after 4 h and cells were further incubated in fresh medium. The expression of plasmid DNA was examined by an epi–fluorescence microscope. To generate stably transfected cells, cells were selected using culture media containing Geneticin (Gibco) at 300–800 μg / ml or Hygromycin B (Invitrogen) at 300 μg / ml over 2 weeks.

### Treatment with pharmacological inhibitor

Taxol (Paclitaxel, Sigma, stock 10 mM in ethanol), Colchicine (Sigma, stock 50 mg/ml in ethanol), ATM inhibitor (Calbiochem, 118,500, stock 10 mM in DMSO) [19] and DNA–PK inhibitor (NU7026, Selleckchem, 324,788, stock 10 mM in DMSO) [20] were applied to cells 45 min prior to radiation exposure at 10 μM, 50 nM and at indicated concentrations, respectively.

### DNA damage induction using x–ray radiation

Cells were trypsinized and transferred either into a falcon tube as suspension culture or onto 35 mm glass bottom culture dishes coated with Poly–D–Lysine (MatTek). Irradiation was delivered while cells were in suspension at room temperature using 6 Gy single doses of 200 kV x–rays (Yxlon Y.TU 320; dose rate∼1.3 Gy/min at 20 mA) filtered with 0.5 mm Cu as published [21]. The absorbed dose was measured using a Duplex dosimeter (PTW).

### Total protein extractions and Western blotting

One day after plating cells onto 6–well plates or at 1 h after radiation exposure, cells were harvested by scraping using cell lysis buffer (Cell Signaling). Total protein concentration (mg/ml) of cell lysate was determined using a spectrophotometry (Nanodrop, Thermo Fisher). Samples were used immediately or stored at − 80 °C until use. Proteins were separated by sodium dodecyl sulfate–polyacrylamide gel electrophoresis (SDS–PAGE) prior to Western blotting. Detection of specific proteins was performed with enhanced chemiluminescence reagent (Amersham) after antibody binding using LAS–3000 imaging system (Fuji).

### Co–culture system and fluorescent staining in living cells

**Fig. 1:**
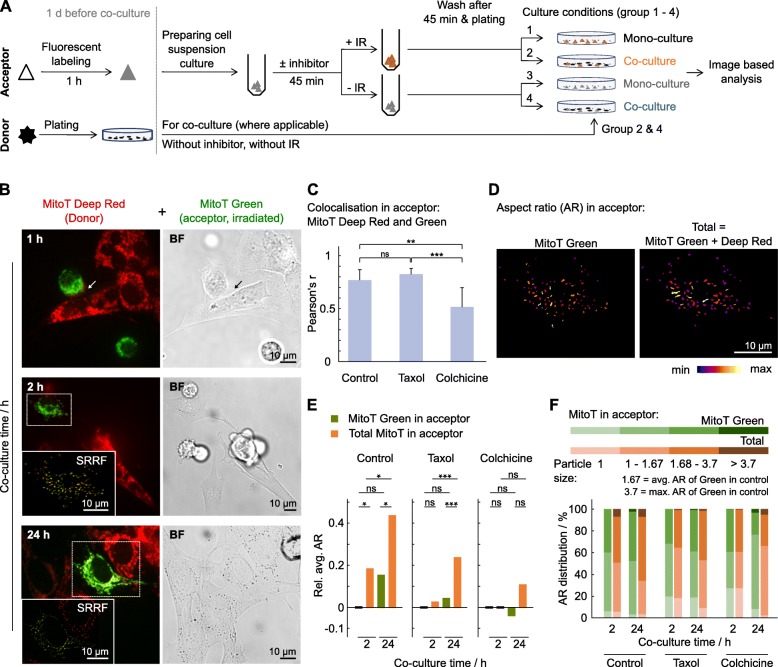
Mitochondria exchange between DNA–damaged and healthy fibroblasts. **A** Experimental setup of co–cultures. IR, ionizing radiation (x–rays). **b** Representative images of mitochondria exchange between unirradiated (MitoT Deep Red) and 6–Gy irradiated (MitoT Green) fibroblasts (arrow: cell–cell contact; white boxed area: Super–resolution radial fluctuation (SRRF) images). **c** Pearson correlation analysis of colocalization between MitoT Deep Red and Green in acceptor cells at 24–h of co–culture under indicated pretreatments. *n* = 30–50 cells. **d** Representative shape description images of aspect ratio (AR) using single cell analysis of acceptor cells at 2–h of co–culture as in **b**. Average AR value of MitoT Green = 1.67 (used as control for **e**), maximum AR = 3.7. **e** Comparison of indicated conditions to 2–h in acceptor of MitoT Green. **f** Size distribution of AR und indicated conditions. Data represent mean ± SD (two–sided t–test; ns, not significant, *P < 0.05, **P < 0.01, ***P < 0.005)

We deployed a co–culture system in which healthy cells served as donors and irradiated DNA–damaged cells as acceptor (Fig. 1a). For microscopic analysis, cells were incubated with 20 μM CellTracker Red CMTPX, 500 nM MitoTracker Red CMXRos or Deep Red FM (Invitrogen) in cell culture flask at 37 °C for 1 h, then carefully washed to remove unbounded dyes one day before co–culture experiments. One hour before co–culture, target cells were trypsinized and transferred as cell suspension with identical cell numbers into two falcon tubes: one for 6–Gy x–ray irradiation; one as unirradiated control. Where indicated, cells were treated with inhibitors for 45 min prior to irradiation. After irradiation, cells were washed and centrifuged at 120 x g for 1 min to remove soluble free. Subsequently, cells were again divided into two groups with identical cell numbers into two falcon tubes. One target cell population was allowed to grow in presence of non–target cells. The other target cell population grew alone. The non–target cell population was plated 24 h prior to the addition of a target cell population at a 70% confluency. Collectively, there are four experimental conditions: i) mono–culture of irradiated target cells, ii) co–culture containing non–target and irradiated target cells, iii) co–culture containing non–target and unirradiated target cells, and iv) mono–culture of unirradiated target cells.

### Immunofluorescence staining

Immunofluorescence (IF) staining was conducted as published [22]. Briefly, cells were fixed with 3.7% paraformaldehyde at room temperature (RT) for 10 min and permeabilized with 0.5% Triton X–100 at RT for 5 min. Then, cells were incubated with blocking buffer (3% BSA in PBS) at RT for 1 h, followed by incubation with primary and secondary antibody in blocking buffer in the dark at RT for 1 h, respectively. The cells were stored in the dark at 4 °C until being imaged.

### Epifluorescence and spinning disc confocal microscopy

All the microscopic experiments were performed in 35 mm culture dishes with glass bottom. For image–based analysis with large cell numbers as shown in Additional file 1: Figure S1, samples were screened by Zeiss Axio Observer Inverted microscope using a 10x/ objective. For confocal microscopy, the images were recorded by spinning disc confocal microscopy (Olympus IX83 microscope with Yokogawa CSU–X1 Confocal Spinning Disc unit, and iXon Ultra 897 EMCCD Camera) with Olympus Plan S Apo 40x/0.95 or 60x/1.2 NA water immersion objective. Confocal fluorescence images were obtained by sequential excitation, if more than two fluorophores were monitored. The fluorescence was measured for DAPI and Hoechst 33342 with excitation wavelength of 405 nm and emission wavelengths 425/45 nm; Alexa 488, MitoTracker Green FM and GFP with excitation wavelength of 488 nm and emission wavelengths 525/20 nm; Alexa 546 and 594, CellTracker Red CMTPX and MitoTracker Red CMXRos with excitation wavelengths of 561 nm and emission wavelengths 617/73 nm; Alexa 633 and 647, DRAQ5 and MitoTracker Deep Red FM with excitation wavelengths of 640 nm and emission 685/40 nm. Phase–contrast images were recorded simultaneously in indicated experiments. Z–stacks with a step of 1 μm were acquired per image.

### Image analysis

All images were processed and analyzed quantitatively using Fiji [23]. Colocalization was based on raw images and analyzed by Pearson correlation analysis using the coloc2 plugin (https://imagej.net/Coloc_2). Images for shape distribution analysis were segmented and analyzed using extensive toolkits VioVoxxel (https://imagej.net/BioVoxxel_Toolbox) [24]. Shape of the particles were represented using aspect ratio, circularity and roundness,

$$ \mathrm{Aspect}\ \mathrm{ratio}=\frac{major\ axis}{minor\ axis} $$

$$ Circularity=\frac{4\pi \times Area}{Perimeter^2} $$

$$ Roundness=\frac{4\times area}{\pi \times {major\ axis}^2} $$

### Super–resolution radial fluctuation analysis

Images for super–resolution radial fluctuation (SRRF) analysis were acquired by spinning disc confocal microscopy as described above. To yield a super–resolution frame, a stack of 200 raw data images were processed by running SRRF program with a ring radius of 1. The SRRF algorithm plugin for Fiji is provided by Gustafsson and colleagues [25].

### Antibodies

Primary antibodies: anti–53BP1 (IF 1:400, WB 1:1000, Novus Biologicals, NB100–904), anti–ATM (WB 1:1000, Cell signaling, 2873), anti–beta actin (WB 1:10,000, Abcam, ab8224), anti–phospho–ATM S1981 (WB 1:500, Rockland, 200–301–400), anti–phospho–DNA–PK S2056 (IF 1:300, Abcam, ab18192), anti–phospho–Histone H2A.X S193 (γH2AX, IF 1:200, anti–rabbit, Cell signaling, 9718 and 1: 300, anti–mouse, Millipore, 05–636), anti–Tubulin (IF 1:500, Abcam, ab6160). Secondary antibodies for IF were purchased from Life Technologies (working dilution at 1:500 in blocking buffer). Those antibodies were validated in the presence or without protein of interest. HRP–conjugated secondary antibodies for WB were from GE Healthcare (working dilution at 1:5000).

### Statistics

All results represent mean ± standard deviation (SD), at least three independent experiments were performed. Unpaired, two–sided Student’s t–test was performed by Microsoft Excel. A *p*–value is less than 0.5 was considered significant.

## Results and discussion

### Bilateral transfer of mitochondria between acceptor and donor cells

To investigate intercellular communication between DNA–damaged and non–damaged cells, we deployed a co–culture system enabling us to explore how healthy cells serve as donors for prosurvival cues for DNA–damaged acceptor cells (Fig. 1a). To test for efficient repair and restoration of damaged mitochondria by functional mitochondria from healthy cells, we tracked mitochondria originating from unirradiated (donor) and irradiated (acceptor) fibroblasts (Fig. 1b and Additional file 1: Figure S1). We discovered an intensive bilateral exchange accompanied by high level of colocalization (Fig. 1c). Disturbing microtubules known to serve as mitochondria carriers by the microtubule–stabilizing agent taxol [26] conserved bilateral mitochondria exchange in contrast to the microtubule polymerization blocker colchicine [27] (Additional file 2: Figure S2). Analyzing mitochondria shapes revealed induced formation of tubular structures (high aspect ratio (AR) value) and fusion between donor and acceptor mitochondria in co–culture (Fig. 1d–f and Additional file 3: Figure S3, Additional file 4: Figure S4). Hence, irradiation–induced fragmentation causative for mitochondria dysfunctionality seemed to be repaired by mitochondria exchange and fusion from healthy donor cells. This process depended on an existing microtubule network as taxol but not colchicine enabled more tubular, less fragmented mitochondria.

Based on intercellular trafficking of DNA repair proteins like ataxia telangiectasia mutated (ATM) along microtubules [28] and ATM involvement in mitochondria homeostasis and redox–sensing [29, 30], we next determined the relevance of ATM for mitochondria exchange upon DNA damage. In contrast to co–cultures of ATM wildtype (wt) fibroblasts (Additional file 5: Figure S5a and b), co–cultures of unirradiated ATM^−/−^ with irradiated ATM^−/−^ fibroblasts lacked mitochondria transfer (Additional file 5: Figure S5f).

ATM–deficient cells are incapable of mitochondria delivery to neighboring cells suggesting mitochondria transfer to follow a one–way, here ATMwt–to–ATM^−/−^ fibroblasts, course of action independent from whether ATM^−/−^ fibroblasts act as acceptor or donor (Additional file 5: Figure S5). The strong spreading ability of MitoT Red has often been observed [31, 32]. However, in our hands, MitoT Red originating from ATM^−/−^ cells could not be found in neighboring cells (Additional file 5: Figure S5 g). We, therefore, carried out further control experiments by using different combinations of MitoT (Additional file 5: Figure S5 g), and, different ratios of donor/acceptor cell numbers (Additional file 5: Figure S5 h). Moreover, the laser intensity for detecting ATM^−/−^ cells was appropriately overexposed. Taken together, our data suggest that ATM functionality seemed to govern the ability for intercellular mitochondria transfer,as one of the foundations for later fusion, independent of the status of damage.

### Accelerated DNA repair through presence of healthy cells

**Fig. 2:**
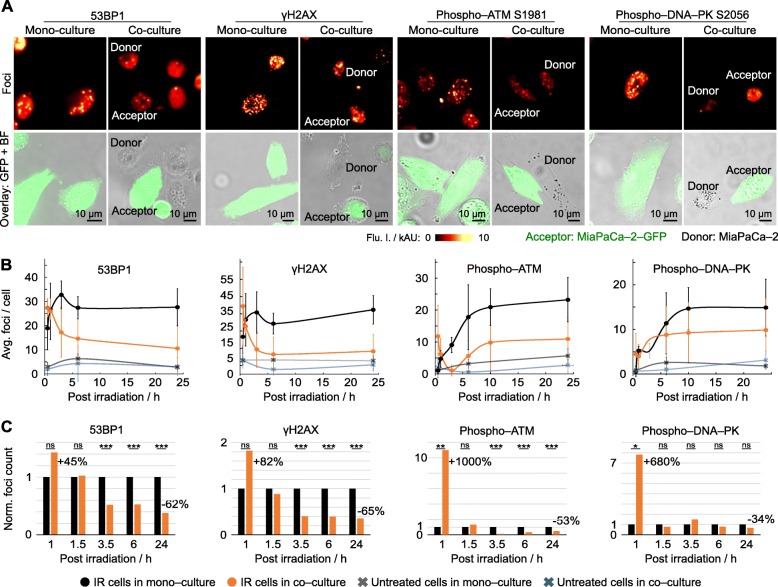
DNA repair is improved by presence of healthy cells. **a** Representative images of maximum intensity projections of indicated radiogenic foci in mono– and co–cultured MiaPaCa–2 cells at 24 h post irradiation (6 Gy x–rays). Foci visualized in acceptor (MiaPaCa–2–GFP; 6–Gy) and donor cells (unlabeled MiaPaCa–2; non–irradiated) by immunofluorescence staining. **b** and **c** Dynamic of radiogenic foci resolution over 24 h in absolute (**b**) and relative numbers (**c**) per irradiated cell (at least 50 cells analysed). Data represent mean ± SD (two–sided t–test; ns, not significant; *P < 0.05, **P < 0.01, ***P < 0.005)

To identify a linkage between mitochondria exchange, DNA repair and cell cycling, we analyzed the repair kinetic of radiogenic DNA double strand breaks (DSB). We commenced the recording of 53BP1, phospho–histone H2AX S193 (γH2AX), phospho–ATM S1981 (pATM) and phospho–DNA–PK S2056 (pDNA–PK) foci 1 h after irradiation, i.e. 0.5 h after seeding, in parallel to cell size in acceptor cells (Fig. 2, Additional file 6: Figure S6 and Additional file 7: Figure S7). While similar degrees of maximal damage were detectable in irradiated mono– and co–cultures (Fig. 2b), the resolution kinetic of 53BP1, γH2AX, pATM and pDNA–PK in acceptor cells presented profoundly and significantly different when co–cultured with healthy cells compared with mono–cultures. Irradiated co–cultures showed pronouncedly elevated foci numbers relative to mono–culture at 1 h after irradiation, which was followed by a strong decline (Fig. 2c and Additional file 6: Figure S6c–f). These results show that DSB repair is remarkably improved by the presence of healthy cells suggesting damaged acceptor cells to receive cytoprotective, DNA repair–modulating signals from undamaged donor cells. In addition to the classic bystander effect [10, 11], our observations demonstrated an untreated-to-irradiated pro-survival signaling.

### Co–culturing modulates cell cycle distribution

We next analyzed simultaneously cell cycling and DSB as tightly and cooperatively intertwined processes [33]. In contrast to mono–culture, the presence of healthy cells resulted in two separable acceptor cell fractions upon DNA damage: G2–phase cells with high and G1–phase cells with low 53BP1 foci numbers (Additional file 8: Figure S8). These data demonstrate minimal differences in cell cycling of DNA damaged cells when co–cultured with healthy cells relative to mono–cultures as well as a cell cycle phase–dependent association with DSB.

### Cell–to–cell signaling stimulated the DNA damage response in ATM deficient cells

**Fig. 3:**
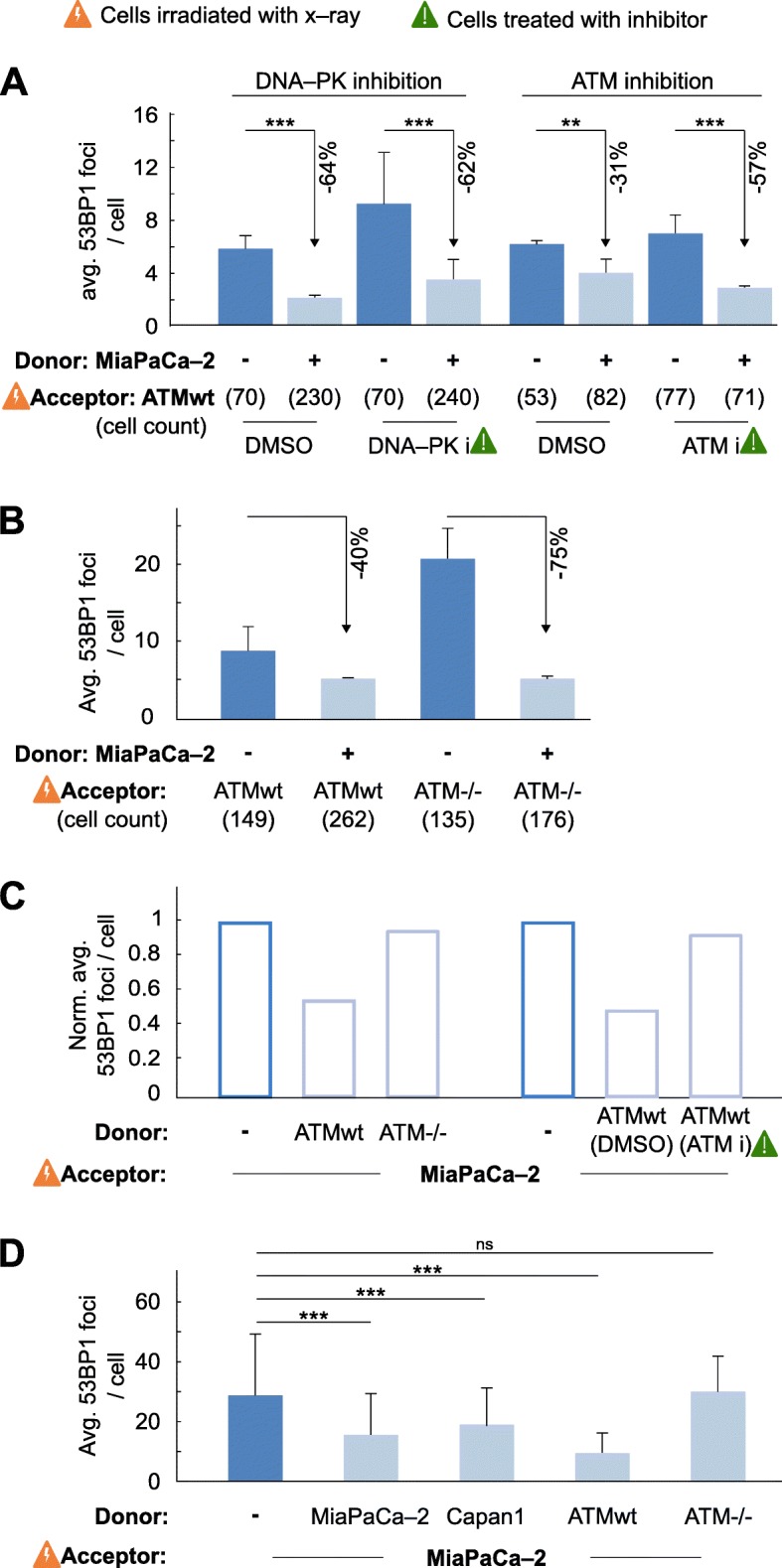
DSB repair improvement by acceptor–donor cell interaction depends on ATM kinase activity. **a** Residual, 24–h 53BP1 foci in ATMwt fibroblasts (acceptor) pretreated with DNA–PK or ATM inhibitors, irradiated with 6–Gy x–rays and subsequently cultured alone or in co–culture with untreated MiaPaCa–2 cells (donor). DMSO used as control. **b** Residual, 24–h 53BP1 foci numbers in 6–Gy irradiated ATMwt or ATM^−/−^ fibroblasts (acceptor) grown in mono– or co–culture with untreated MiaPaCa–2 cells (donor). **c** Normalized residual 53BP1 foci numbers in 6–Gy irradiated MiaPaCa–2 cells (acceptor) in mono– or co–culture with either ATMwt or ATM^−/−^ fibroblasts (donor) or ATMwt fibroblasts pretreated with ATM inhibitor (DMSO used as control). **d** Comparison of 53BP1 foci numbers in 6–Gy irradiated MiaPaCa–2 cells (acceptor) cultured either alone or in co–culture with untreated MiaPaCa–2 cells, Capan1 cells, ATMwt or ATM^−/−^ fibroblasts (donors). Number of analysed irradiated acceptor cells is n = 30, unless otherwise indicated. Data represent mean ± SD (two–sided t–test; ns, not significant, **P < 0.01, ***P < 0.005)

To further determine the underlying mechanisms of enhanced DSB repair in co–cultured cells, we pharmacologically inhibited DNA–PK and ATM to discriminate between their pathways (Additional file 9: Figure S9). In line with previous reports on impaired DNA repair under inhibition [19, 20], we observed a significant, foci reduction of 57% in ATMwt co–cultures than mono–cultures (31%) upon ATM inhibition (Fig. 3a and Additional file 10: Figure S10). Foci reduction upon DNA–PK inhibition was, however, similar in co– and mono–cultures (both appr. 60%). Confirmatory data were generated in another cell model with ATMwt or ATM^−/−^ (Additional file 11: Figure S11) fibroblasts as acceptors co–cultured with human MiaPaCa–2 pancreatic cancer cells showing a 75% versus 40% decline of 53BP1 foci in ATM^−/−^ versus ATMwt acceptor fibroblasts 24 h after irradiation (Fig. 3b). These observations indicate ATM to be critically involved in DSB repair of acceptor cell populations in presence of healthy donor cell populations.

### ATM deficient cells are incapable of rescuing DNA damaged neighboring cells

Next, we examined whether ATM signaling is fundamental in healthy donors for rescuing damaged neighboring cells. A functional ATM status determined an appr. 50% DSB reduction in irradiated MiaPaCa–2 cells co–cultured with healthy, ATM–competent fibroblasts relative to mono–culture, co–culture with ATM–incompetent fibroblasts or pharmacologically inhibited ATM (Fig. 3c). As ATM activates both error–prone NHEJ and error–free HR repair [34], we employed NHEJ/HR–proficient (MiaPaCa–2, ATMwt fibroblasts), NHEJ–deficient (ATM^−/−^ fibroblasts) and HR–deficient cells (BRCA2–depleted Capan1) as donors and found that, when co–cultured, NHEJ/HR–proficient MiaPaCa–2 acceptor cells show optimized DSB repair dependent on NHEJ and functional ATM but not HR (Fig. 3d). These observations suggest a prominent role of ATM for donor–to–acceptor but not acceptor–to–donor cell signaling to provide specific, yet to be identified cues, for a more effective NHEJ–related DSB repair.

### Intercellular signaling requires microtubule polymerization

**Fig. 4:**
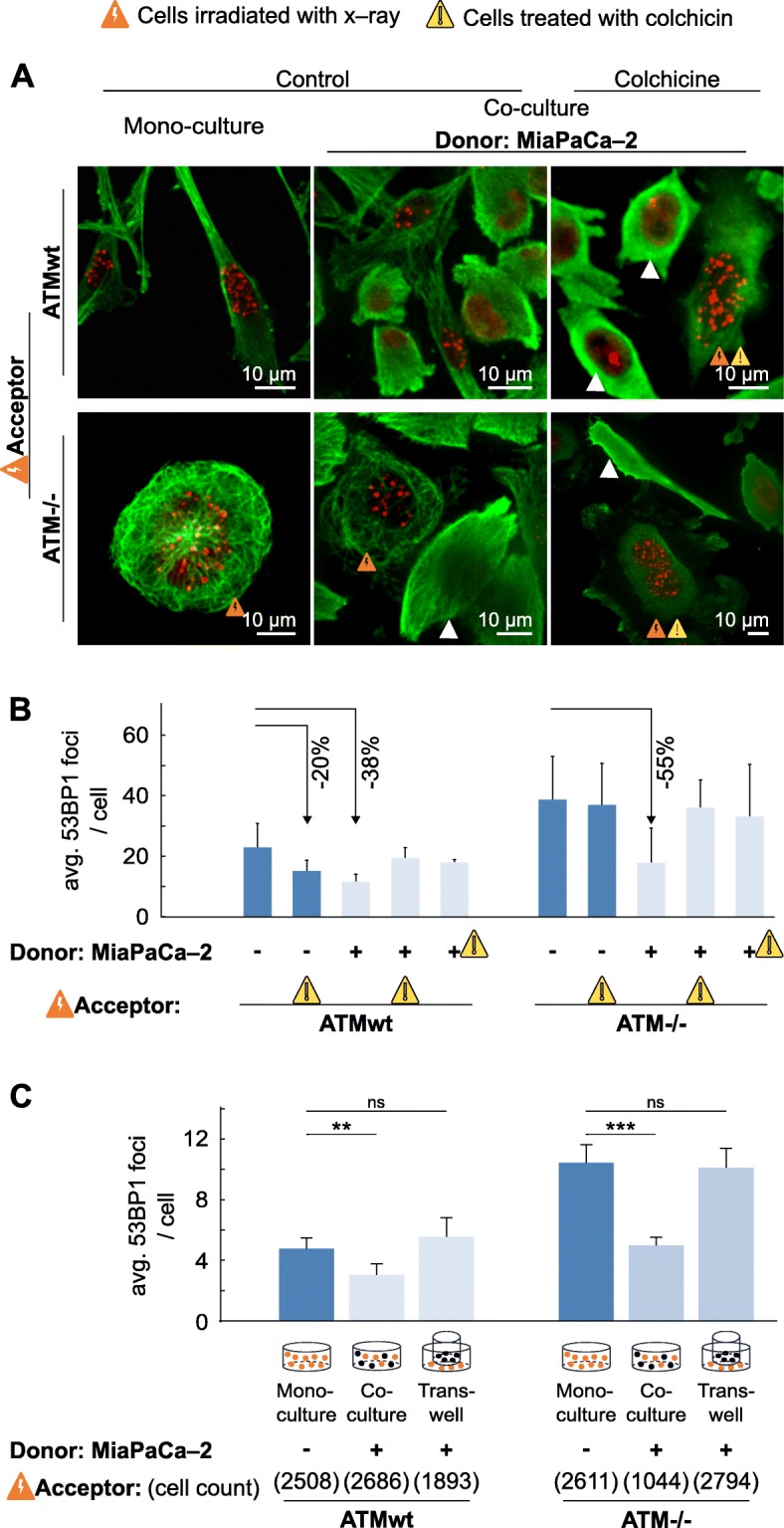
DSB repair depends on microtubule networks. **a** Representative maximum intensity projections of whole z–stacks visualizing 53BP1 foci in ATMwt or ATM^−/−^ fibroblasts and microtubules using immunofluorescence staining. Untreated, irradiated or colchicine–pretreated fibroblasts (acceptor) were either plated for mono– or co–culture together with MiaPaCa–2 cells (donor; white triangles). **b** Residual, 24–h 53BP1 foci analyses from 6–Gy x–rays irradiated fibroblasts (acceptor) with or without untreated or colchicine–pretreated MiaPaCa–2 cells (donor). **c** Residual, 24–h 53BP1 foci analyses from 6–Gy x–rays irradiated fibroblasts (acceptor) in mono–culture, co–culture or transwell (0.4 μm pore size) with MiaPaCa–2 cells (donor). Data represent mean ± SD (*n* = 20–70 irradiated acceptor cells)

Changes in DSB numbers already occurred very early on, at 0.5 h after plating, suggesting a promptness of physical cell–to–cell interaction allowing transfer of stimulatory DNA repair cues. We hypothesized this physical pathway to be, for example, cell connecting, tubulin–based membrane tubes as demonstrated by others using different approaches and cell culture systems [28, 35, 36]. In mono–culture, inhibited microtubule polymerization, generated by colchicine in donor or acceptor cells (Fig. 4a and Additional file 12: Figure S12), elicited 20% less foci numbers in ATMwt in contrast to ATM^−/−^ fibroblasts compared to their respective controls (Fig. 4b). In co–culture, colchicine impaired improvement of DSB repair in acceptor cells independent from which side, i.e. acceptor or donor, the microtubule network was disturbed. These results indicate the necessity of a functional microtubule system for transfer of cytoprotective cues between DNA–damaged and undamaged cell populations. Moreover, as additional control, culturing donor and acceptor cells in the the same cell culture medium but separating them in space by using a transwell system (0.4 μm pore size), the average 53BP1 foci numbers in acceptor cells are similar to those detected in mono–cultures (Fig. 4c). This indicates that a direct contact between donor and acceptor cells via, e.g. gap junctions and/or membrane nanotubes, promotes this type of healthy–to–damaged cell prosurvival signaling.

## Conclusion

Cells communicate constantly with their environment including neighboring cells as well the extracellular matrix by giving and receiving signals. Studies over the past decades have provided ample evidence on how unirradiated cells are damaged through communication with neighboring irradiated cells. Very little attention, however, has been paid on the reverse, i.e. feedback signals from healthy cells to DNA damaged cells. The prosurvival and DNA repair modulating effects transferred from undamaged to damaged cells are largely unknown. For both normal and cancer tissue, it would be fundamental to clarify whether this feedback exists, especially when target cell signals fail to trigger a DNA damage response in non–target cells, what are such signals, how are these signals delivered to target cells and what is the response pattern of target cells.

Here, we addressed the DNA damage response in genotoxically injured cells cultured in the presence or absence of undamaged cells. Collectively, our data show that (i) bilateral transfer of mitochondria between target (irradiated) and non–target cells occurs in an ATM–dependent manner; (ii) DNA damage repair in target (irradiated) cells is accelerated through the presence of non–target cells in both ATMwt and ATM deficient fibroblasts; (iii) cell cycle distribution in target cells is substantially different in co–cultured target cells as compared to mono–cultures of target cells; and (iv) a functional microtubules system is required for this intercellular signaling through direct cell–to–cell contact.

These findings demonstrate that cell–cell communication between DNA–damaged and healthy cells provides effective and immediate prosurvival feedback. The hierarchical organization between microtubules, mitochondria and ATM pinpoints the complexity of such regulatory networks allowing multiple ways for rescue and evasion. While this is fundamental for normal tissue integrity, rescue signals may particularly be considered in various diseases such as cancer, in which inherent and acquired resistance mechanisms to therapies impede the cure of patients. Given the interactive and regulatory role of these three target structures, it seems promising to search for combinatorial therapy approaches that abrogate prosurvival feedback between damaged and undamaged cell populations.

## Supplementary information

**Additional file 1: Figure S1.** Mitochondria exchange between x–ray–damaged and undamaged fibroblasts. Corresponding to Fig. 1b. Representative images of mitochondria exchange between unirradiated (MitoT Deep Red) and 6–Gy irradiated (MitoT Green) fibroblasts at 0.5 h (**a**), 1 h (**b**), 2 h (**c**) and 24 h (**d**) co–culture time. Super–resolution radial fluctuation (SRRF) images and colour maps of aspect ratio (AR) of white boxed area in c and d. Scale bars, 10 μm.

**Additional file 2: Figure S2.** Mitochondria exchange between undamaged and x–ray–damaged, drug pretreated fibroblasts. Representative super–resolution radial fluctuation (SRRF) images and AR colour maps of mitochondria exchange between unirradiated (MitoT Deep Red) and 6–Gy irradiated (MitoT Green) fibroblasts, which were pretreated with taxol (a and b) or colchicine (c and d), at 2 h and 24 h co–culture time. Scale bars, 10 μm.

**Additional file 3: Figure S3.** Analysis of mitochondrial morphology. **a** Representative image of mitochondria networks labeled with MitoT Deep Red in untreated fibroblast. **b** Segmentation of white boxed area in a. **c** Colour maps of aspect ratio (AR), circularity and roundness as in b. **d** Mitochondria exchange between unirradiated (MitoT Deep Red) and 6–Gy irradiated (MitoT Green) fibroblasts, under untreated, taxol and colchicine conditions. **e** Single cell analyses of mitochondria shapes of MitoT Green from acceptor cells in d.

**Additional file 4: Figure S4. a** Mitochondria exchange between DNA–damaged and healthy fibroblasts. Corresponding to Fig. 1e. The absolute values of average aspect ratios (avg. AR). b Comparison of indicated conditions to 2–h control. Results represent average AR–values of 30 cells ± SD (two–sided t–test; ns, not significant, *P < 0.05, **P < 0.01, ***P < 0.005).

**Additional file 5: Figure S5. a–f** Mitochondria transfer in ATMwt and ATM^−/−^ fibroblasts upon irradiation. Mitochondria transfer was monitored between donor cells labeled with MitoTracker Deep Red (green, indicated with white marker) and 6–Gy irradiated acceptor cells labeled with MitoTracker Red (red, indicated with orange marker) after 24 h of co–culture. Nuclei were stained with DAPI. Co–culture of ATMwt and irradiated ATMwt fibroblasts (a and b), ATMwt and irradiated ATM^−/−^ fibroblasts (c and d), ATM^−/−^ and irradiated ATMwt fibroblasts (e), as well ATM^−/−^ and irradiated ATM^−/−^ fibroblasts (f). g Unilateral transfer of mitochondria from irradiated ATMwt (labeled with MitoTracker Deep Red) to ATM^−/−^ fibroblasts (labeled with MitoTracker Green, indicated with white marker). h Unilateral transfer of mitochondria from ATMwt (labeled with MitoTracker Deep Red, indicated with white marker) to irradiated ATM^−/−^ fibroblasts (labeled with MitoTracker Red). SRRF: super–resolution radial fluctuation images. Scale bars, 10 μm.

**Additional file 6: Figure S6.** Dynamics of foci resolution in mono– and co–cultured irradiated cells. **a** Corresponding to Fig. 2a. Overlay images show the nucleus location of foci detected by IF. Images were acquired by spinning disc confocal microscopy using a 40x objective. Scale bars, 10 μm. **b** Cell size dynamics of 6–Gy irradiated and non–irradiated, mono– and co–cultured acceptor cells over a time interval of 24 h. Related to Fig. 2b–c. **c–f** Resolution dynamics of 53BP1 (**c, d**) and phospho–ATM S1981 (**e, f**) foci. Foci were visualized by IF, imaged by epi–fluorescence microscopy using a 10x objective. *n* = 300 (at first time point) to 5000 (at last time point). **g** Reduction of 53BP1 foci number in acceptor cells depends on ratio of donor–to–acceptor cell numbers. Results represent mean ± SD (two–sided t–test; ns, not significant, *P < 0.05, **P < 0.01, ***P < 0.005).

**Additional file 7: Figure S7.** Co–localization of γH2AX and 53BP1 foci. 6–Gy irradiated MiaPaCa–2–GFP cells (acceptor, in green) in mono– and co–culture with untreated MiaPaCa–2 (donor) 24 h after plating. IF images show co–localization of γH2AX and 53BP1 foci. Scale bars, 10 μm.

**Additional file 8: Figure S8.** Co–culture conditions profoundly change the association between DSB repair and cell cycling. **a** Schematic of the Fucci system. **b** Representative images of MiaPaCa–2 acceptor cells transfected with the Fucci system to monitor G1 (red) and G2 (green) phases. Transfected cells were exposed to 6 Gy x–ray and subsequently plated for either mono– or co–culture together with untreated MiaPaCa–2 cells. After 24 h, 53BP1 foci were determined. In overlay pictures, the first number indicates the ratio of G1 to G2 fluorescence intensity and the second number indicates the foci number in each nucleus. Scale bars, 10 μm. **c** Plot of fluorescence intensity values for G2 vs G1 in each analysed cell. Size of circle radius represents number of foci. **d** Plot of G1 to G2 ratio over number of 53BP1 foci. Bars represent single cell. **e** Proportion of cell numbers in different cell cycle phases.

**Additional file 9: Figure S9.** DSB repair is significantly influenced by acceptor cell/donor cell interactions in an ATM activity–dependent manner. **a** ATMwt fibroblasts were treated with DNA–PK inhibitor (DNA–PK i) and 6 Gy x–rays. Phospho–DNA PK S2056 foci were determined at 1 h post irradiation by immunofluorescence microscopy. DMSO used as control. n indicates cell numbers. **b** Western blot of ATM and pATM (S1981) expression in whole cell lysates from ATMwt fibroblast upon 0 or 6 Gy x–rays irradiation plus/minus exposure to ATM inhibitor. DMSO used a control. Beta actin was used as loading control. **c** Corresponding densitometric analysis of b. **d** Corresponding densitometric analysis of Fig. 3a, including untreated controls (n.c.). Data represent mean ± SD (two–sided t–test; ns, not significant, *P < 0.05, **P < 0.01, ***P < 0.005).

**Additional file 10: Figure S10.** Combination of DNA–PK or ATM inhibitor treatment and irradiation in mono– and co–culture. DNA–PK or ATM inhibitor treated, 6–Gy irradiated ATMwt fibroblasts (acceptor) cultured alone or in co–culture with untreated MiaPaCa–2 cells (donor). Residual, 24–h 53BP1 foci in irradiated ATMwt acceptor cells are analysed. Relative 53BP1 foci values are displayed, corresponding to Fig. 3a.

**Additional file 11: Figure S11.** Expression of ATM, phospho–ATM S1981 and 53BP1 in ATMwt and ATM^−/−^ fibroblasts. Western blot of protein expression in whole cell lysates from 6–Gy x–ray irradiated or unirradiated ATMwt and ATM^−/−^ fibroblasts after 24 h. Beta actin was used as loading control.

**Additional file 12: Figure S12.** DSB repair depends on microtubule networks. **a** Representative images showing immunofluorescence staining of microtubules in MiaPaCa–2 cells, ATMwt and ATM^−/−^ fibroblasts treated with 30 μM colchicine or left untreated. **b** Representative high–resolution image of untreated and colchicine treated cells in co–culture. **c–d** Corresponding to Fig. 4a. Irradiated ATMwt and ATM^−/−^ fibroblasts with or without colchicine pretreatment were either plated for mono– or co–culture together with untreated MiaPaCa–2 cells (white triangles). Representative maximum intensity projections of z–stacks visualizing 53BP1 foci (anti–53BP1 / Alexa594) in ATMwt or ATM^−/−^ fibroblasts and microtubules (anti–tubulin / A405) using immunofluorescence staining. Scale bars, 10 μm.

## Data Availability

All data used in this study are available from the corresponding author on reasonable requests.

## Abbreviations

AR: Aspect ratio
ATM: Ataxia–telangiectasia mutated
DSB: DNA double–strand breaks
HR: Homologous recombination
MitoT: MitoTracker
NHEJ: Non–homologous end joining
NO: Nitric oxide
ROS: Reactive oxygen species
SRRF: Super–resolution radial fluctuation
