# Supplementary figures and images for: ATM controls DNA repair and mitochondria transfer between neighboring cells
Source: Cell Commun Signal. 2019 Nov 8;17:144. doi: 10.1186/s12964-019-0472-x (PMC6842230; doi:10.1186/s12964-019-0472-x)

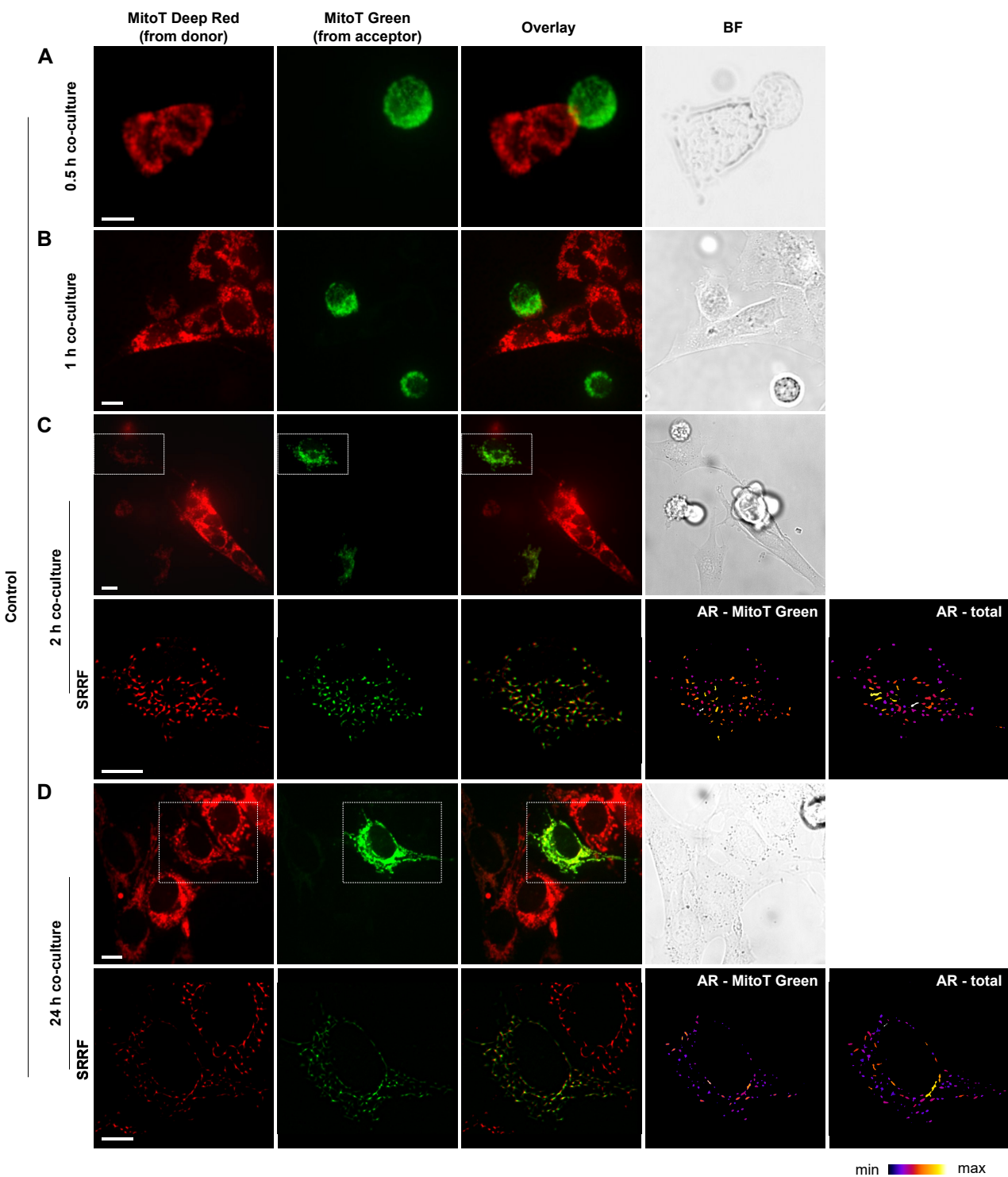

Supplement: Supplementary file 1 — Additional file 1: Figure S1. Mitochondria exchange between x–ray–damaged and undamaged fibroblasts. Corresponding to Fig. 1b. Representative images of mitochondria exchange between unirradiated (MitoT Deep Red) and 6–Gy irradiated (MitoT Green) fibroblasts at 0.5 h (a), 1 h (b), 2 h (c) and 24 h (d) co–culture time. Super–resolution radial fluctuation (SRRF) images and colour maps of aspect ratio (AR) of white boxed area in c and d. Scale bars, 10 μm. [file 12964_2019_472_MOESM1_ESM.pdf]

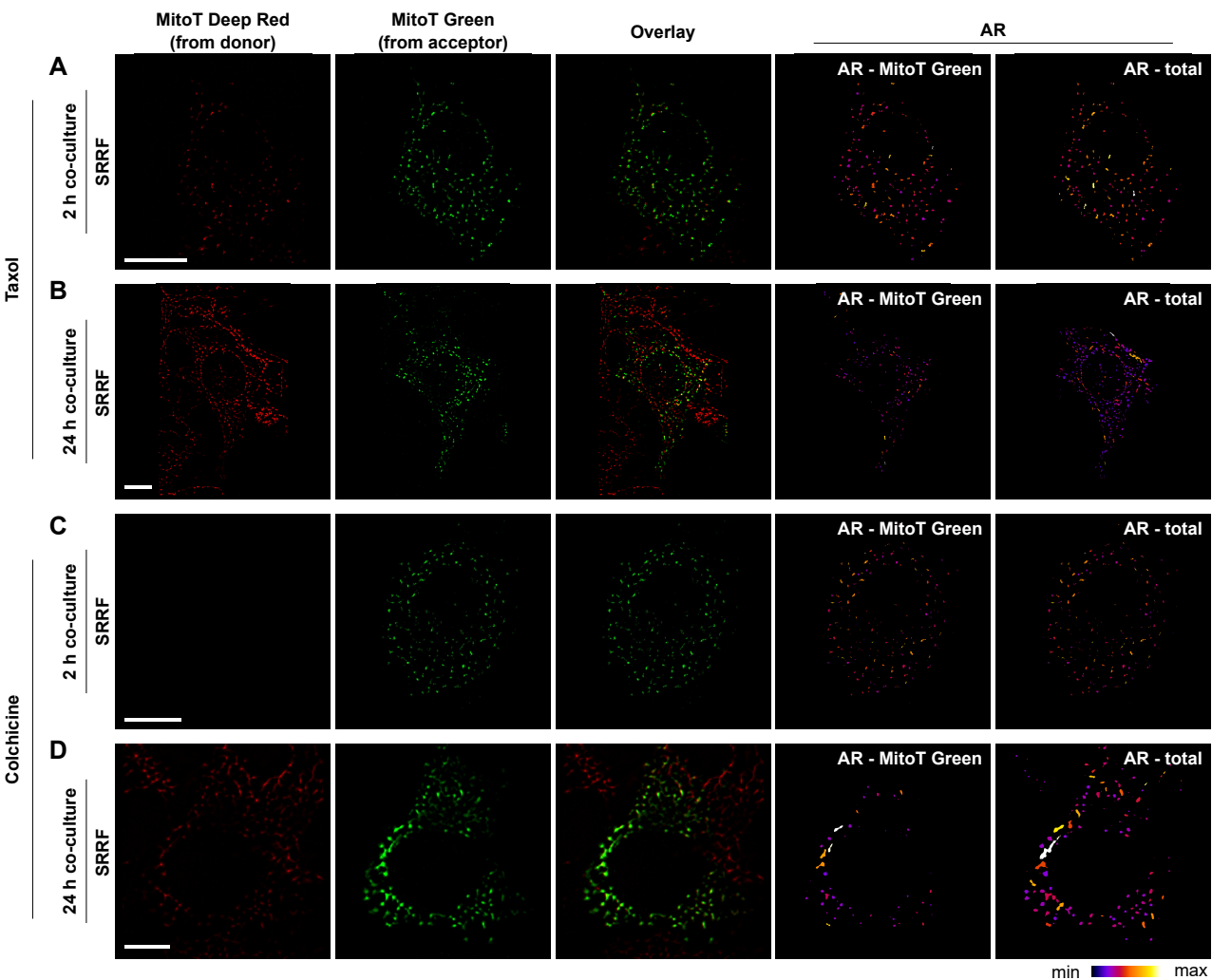

Supplement: Supplementary file 2 — Additional file 2: Figure S2. Mitochondria exchange between undamaged and x–ray–damaged, drug pretreated fibroblasts. Representative super–resolution radial fluctuation (SRRF) images and AR colour maps of mitochondria exchange between unirradiated (MitoT Deep Red) and 6–Gy irradiated (MitoT Green) fibroblasts, which were pretreated with taxol (a and b) or colchicine (c and d), at 2 h and 24 h co–culture time. Scale bars, 10 μm. [file 12964_2019_472_MOESM2_ESM.pdf]

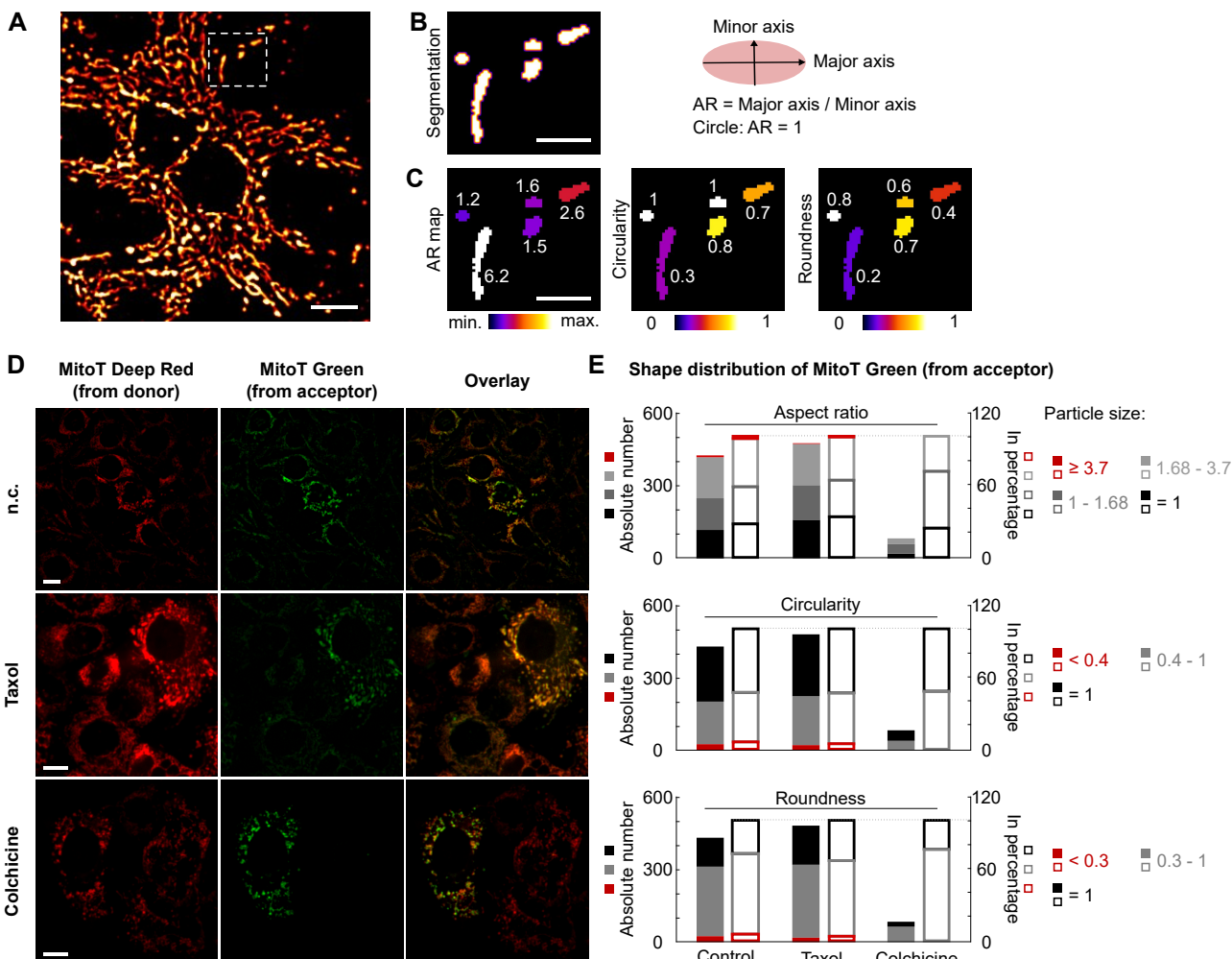

Supplement: Supplementary file 3 — Additional file 3: Figure S3. Analysis of mitochondrial morphology. a Representative image of mitochondria networks labeled with MitoT Deep Red in untreated fibroblast. b Segmentation of white boxed area in a. c Colour maps of aspect ratio (AR), circularity and roundness as in b. d Mitochondria exchange between unirradiated (MitoT Deep Red) and 6–Gy irradiated (MitoT Green) fibroblasts, under untreated, taxol and colchicine conditions. e Single cell analyses of mitochondria shapes of MitoT Green from acceptor cells in d. [file 12964_2019_472_MOESM3_ESM.pdf]

**A**

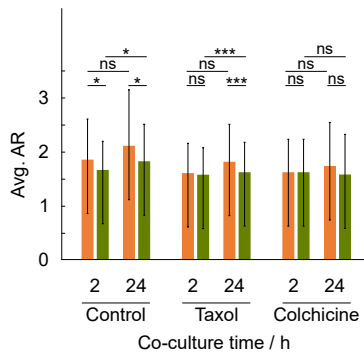

**B**

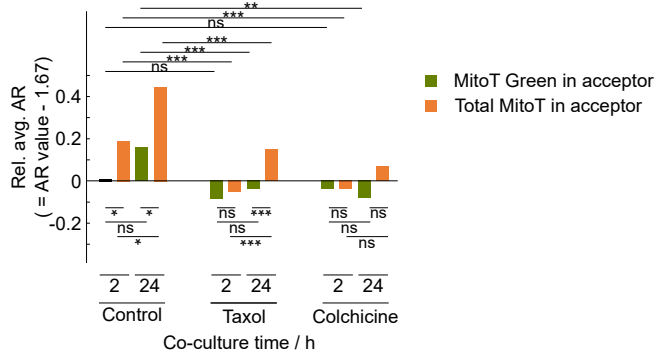

Supplement: Supplementary file 4 — Additional file 4: Figure S4. a Mitochondria exchange between DNA–damaged and healthy fibroblasts. Corresponding to Fig. 1e. The absolute values of average aspect ratios (avg. AR). b Comparison of indicated conditions to 2–h control. Results represent average AR–values of 30 cells ± SD (two–sided t–test; ns, not significant, *P < 0.05, **P < 0.01, ***P < 0.005). [file 12964_2019_472_MOESM4_ESM.pdf]

**G**

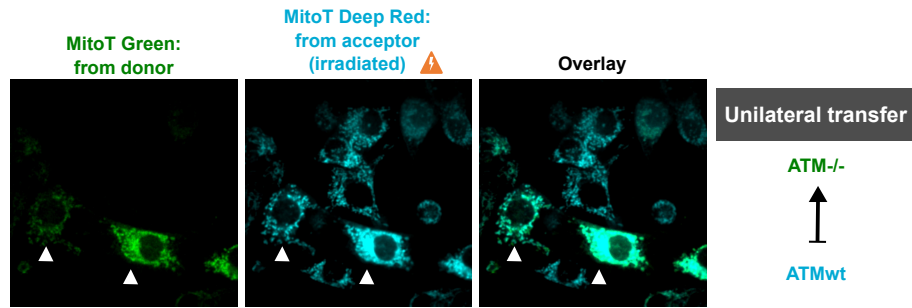

**H**

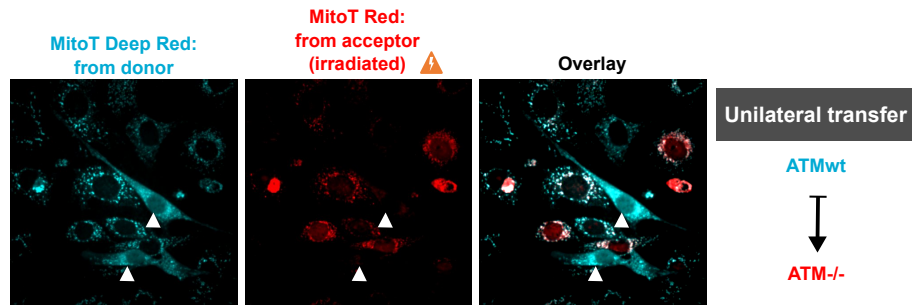

Supplement: Supplementary file 5 — Additional file 5: Figure S5. a–f Mitochondria transfer in ATMwt and ATM−/− fibroblasts upon irradiation. Mitochondria transfer was monitored between donor cells labeled with MitoTracker Deep Red (green, indicated with white marker) and 6–Gy irradiated acceptor cells labeled with MitoTracker Red (red, indicated with orange marker) after 24 h of co–culture. Nuclei were stained with DAPI. Co–culture of ATMwt and irradiated ATMwt fibroblasts (a and b), ATMwt and irradiated ATM−/− fibroblasts (c and d), ATM−/− and irradiated ATMwt fibroblasts (e), as well ATM−/− and irradiated ATM−/− fibroblasts (f). g Unilateral transfer of mitochondria from irradiated ATMwt (labeled with MitoTracker Deep Red) to ATM−/− fibroblasts (labeled with MitoTracker Green, indicated with white marker). h Unilateral transfer of mitochondria from ATMwt (labeled with MitoTracker Deep Red, indicated with white marker) to irradiated ATM−/− fibroblasts (labeled with MitoTracker Red). SRRF: super–resolution radial fluctuation images. Scale bars, 10 μm. [file 12964_2019_472_MOESM5_ESM.zip › Figure S5.1.pdf]

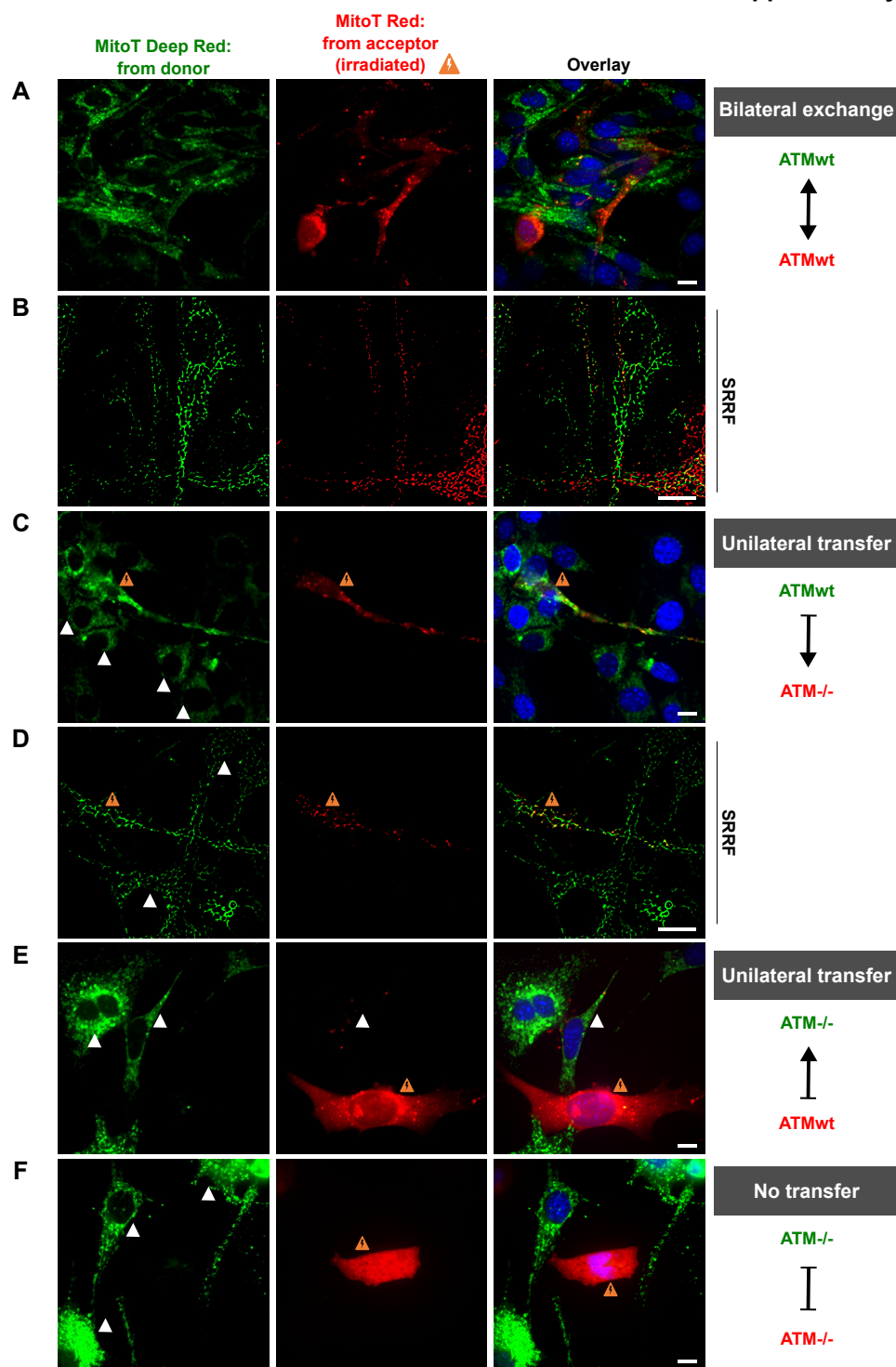

Supplement: Supplementary file 5 — Additional file 5: Figure S5. a–f Mitochondria transfer in ATMwt and ATM−/− fibroblasts upon irradiation. Mitochondria transfer was monitored between donor cells labeled with MitoTracker Deep Red (green, indicated with white marker) and 6–Gy irradiated acceptor cells labeled with MitoTracker Red (red, indicated with orange marker) after 24 h of co–culture. Nuclei were stained with DAPI. Co–culture of ATMwt and irradiated ATMwt fibroblasts (a and b), ATMwt and irradiated ATM−/− fibroblasts (c and d), ATM−/− and irradiated ATMwt fibroblasts (e), as well ATM−/− and irradiated ATM−/− fibroblasts (f). g Unilateral transfer of mitochondria from irradiated ATMwt (labeled with MitoTracker Deep Red) to ATM−/− fibroblasts (labeled with MitoTracker Green, indicated with white marker). h Unilateral transfer of mitochondria from ATMwt (labeled with MitoTracker Deep Red, indicated with white marker) to irradiated ATM−/− fibroblasts (labeled with MitoTracker Red). SRRF: super–resolution radial fluctuation images. Scale bars, 10 μm. [file 12964_2019_472_MOESM5_ESM.zip › Figure S5.pdf]

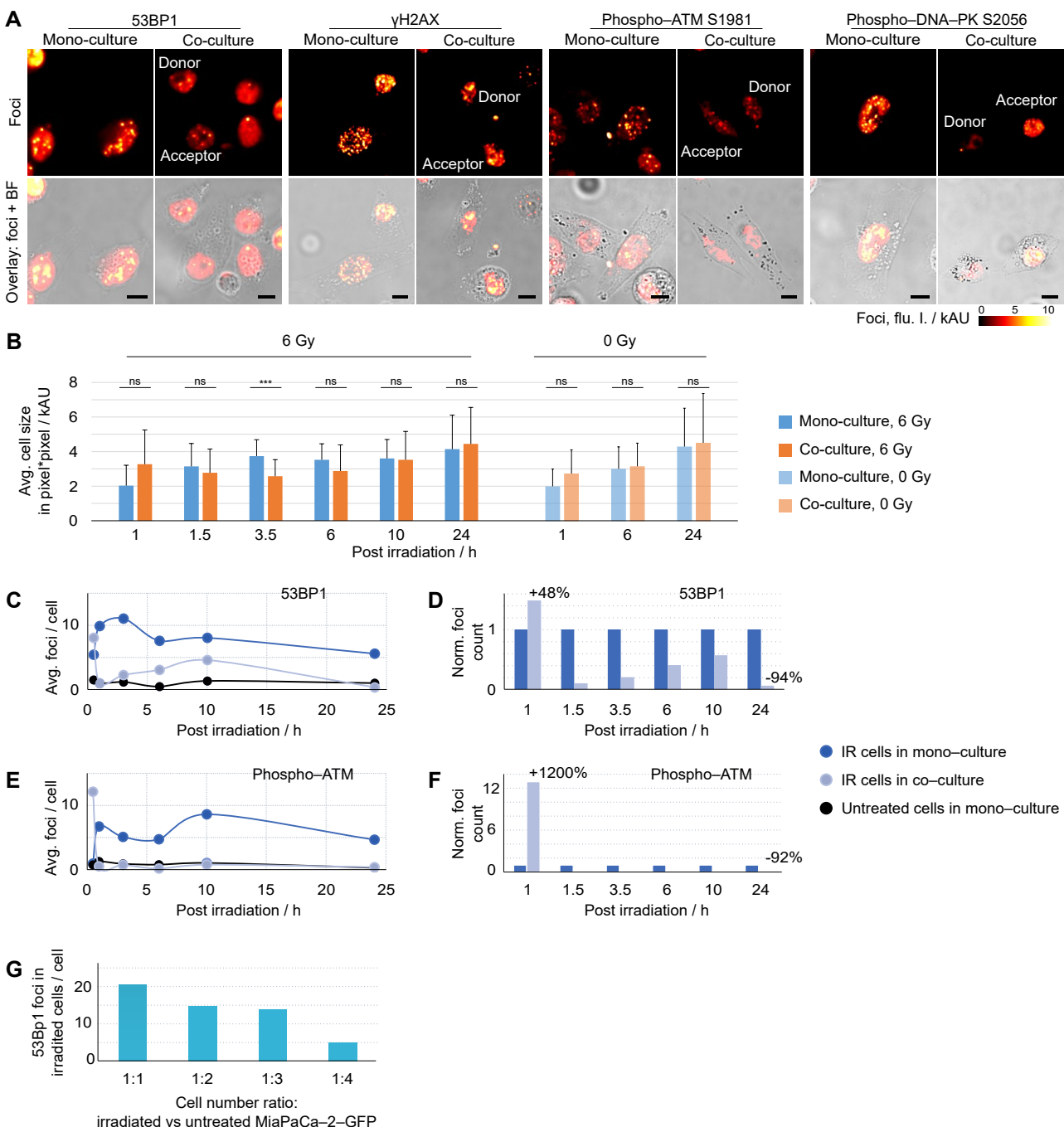

Supplement: Supplementary file 6 — Additional file 6: Figure S6. Dynamics of foci resolution in mono– and co–cultured irradiated cells. a Corresponding to Fig. 2a. Overlay images show the nucleus location of foci detected by IF. Images were acquired by spinning disc confocal microscopy using a 40x objective. Scale bars, 10 μm. b Cell size dynamics of 6–Gy irradiated and non–irradiated, mono– and co–cultured acceptor cells over a time interval of 24 h. Related to Fig. 2b–c. c–f Resolution dynamics of 53BP1 (c, d) and phospho–ATM S1981 (e, f) foci. Foci were visualized by IF, imaged by epi–fluorescence microscopy using a 10x objective. n = 300 (at first time point) to 5000 (at last time point). g Reduction of 53BP1 foci number in acceptor cells depends on ratio of donor–to–acceptor cell numbers. Results represent mean ± SD (two–sided t–test; ns, not significant, *P < 0.05, **P < 0.01, ***P < 0.005). [file 12964_2019_472_MOESM6_ESM.pdf]

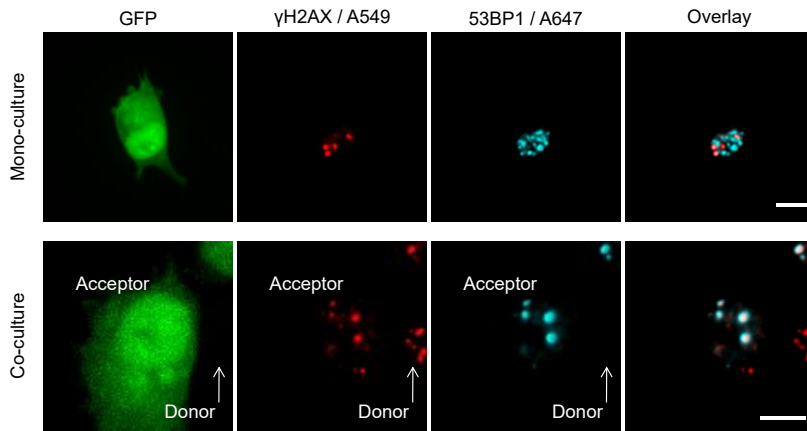

Supplement: Supplementary file 7 — Additional file 7: Figure S7. Co–localization of γH2AX and 53BP1 foci. 6–Gy irradiated MiaPaCa–2–GFP cells (acceptor, in green) in mono– and co–culture with untreated MiaPaCa–2 (donor) 24 h after plating. IF images show co–localization of γH2AX and 53BP1 foci. Scale bars, 10 μm. [file 12964_2019_472_MOESM7_ESM.pdf]

Supplementary Figure 8

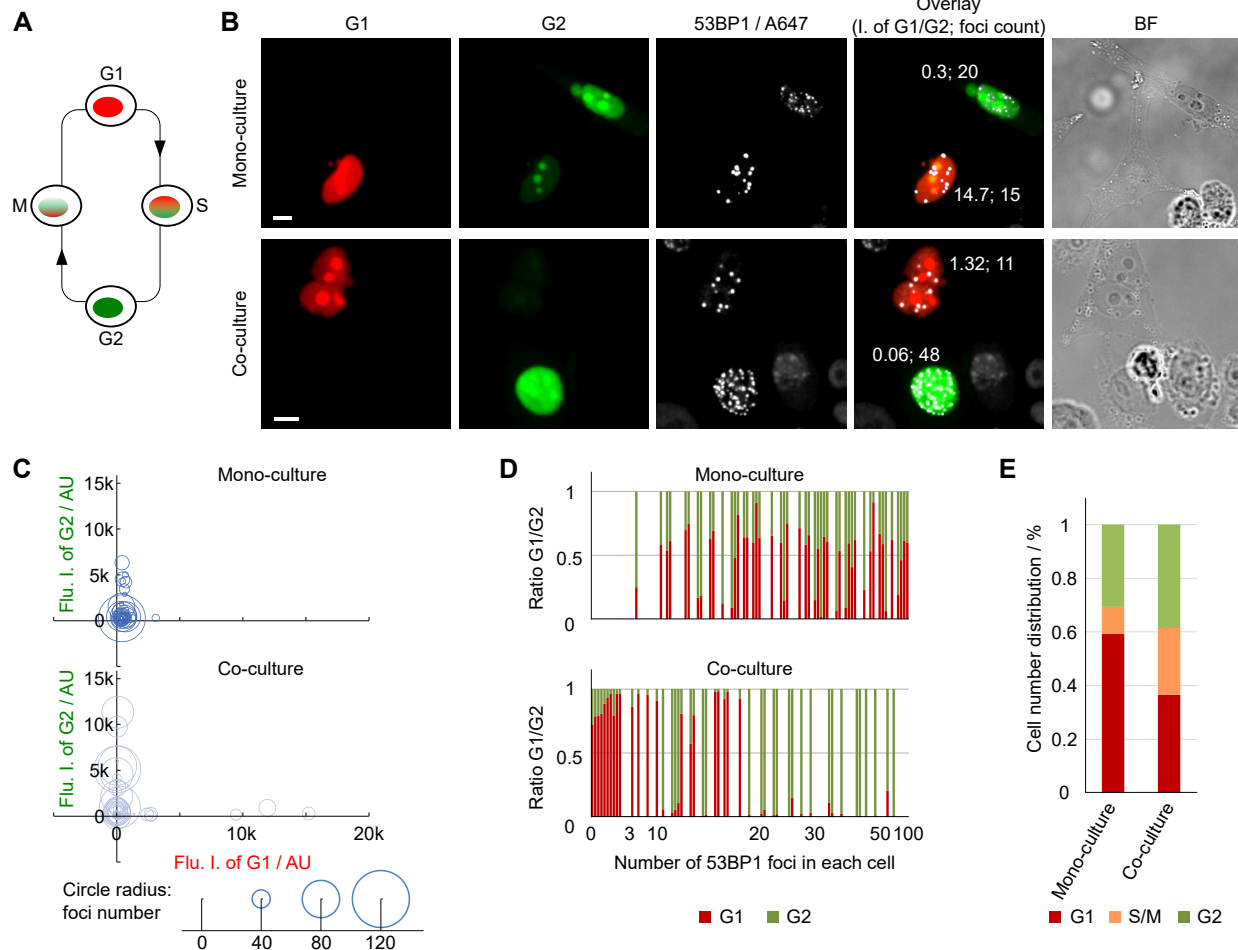

Supplement: Supplementary file 8 — Additional file 8: Figure S8. Co–culture conditions profoundly change the association between DSB repair and cell cycling. a Schematic of the Fucci system. b Representative images of MiaPaCa–2 acceptor cells transfected with the Fucci system to monitor G1 (red) and G2 (green) phases. Transfected cells were exposed to 6 Gy x–ray and subsequently plated for either mono– or co–culture together with untreated MiaPaCa–2 cells. After 24 h, 53BP1 foci were determined. In overlay pictures, the first number indicates the ratio of G1 to G2 fluorescence intensity and the second number indicates the foci number in each nucleus. Scale bars, 10 μm. c Plot of fluorescence intensity values for G2 vs G1 in each analysed cell. Size of circle radius represents number of foci. d Plot of G1 to G2 ratio over number of 53BP1 foci. Bars represent single cell. e Proportion of cell numbers in different cell cycle phases. [file 12964_2019_472_MOESM8_ESM.pdf]

**A**

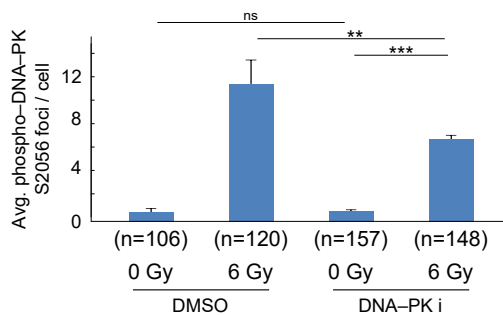

**B**

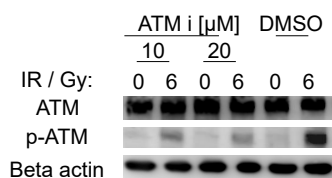

**C**

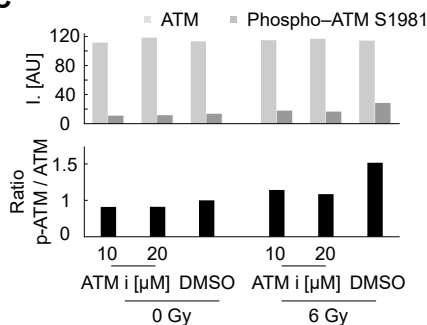

**D**

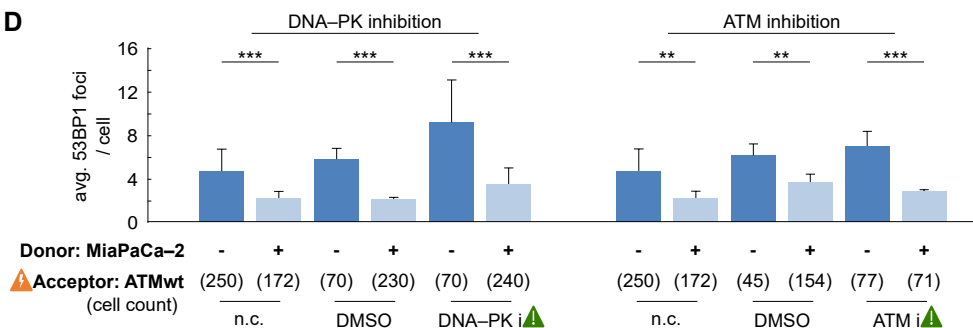

Supplement: Supplementary file 9 — Additional file 9: Figure S9. DSB repair is significantly influenced by acceptor cell/donor cell interactions in an ATM activity–dependent manner. a ATMwt fibroblasts were treated with DNA–PK inhibitor (DNA–PK i) and 6 Gy x–rays. Phospho–DNA PK S2056 foci were determined at 1 h post irradiation by immunofluorescence microscopy. DMSO used as control. n indicates cell numbers. b Western blot of ATM and pATM (S1981) expression in whole cell lysates from ATMwt fibroblast upon 0 or 6 Gy x–rays irradiation plus/minus exposure to ATM inhibitor. DMSO used a control. Beta actin was used as loading control. c Corresponding densitometric analysis of b. d Corresponding densitometric analysis of Fig. 3a, including untreated controls (n.c.). Data represent mean ± SD (two–sided t–test; ns, not significant, *P < 0.05, **P < 0.01, ***P < 0.005). [file 12964_2019_472_MOESM9_ESM.pdf]

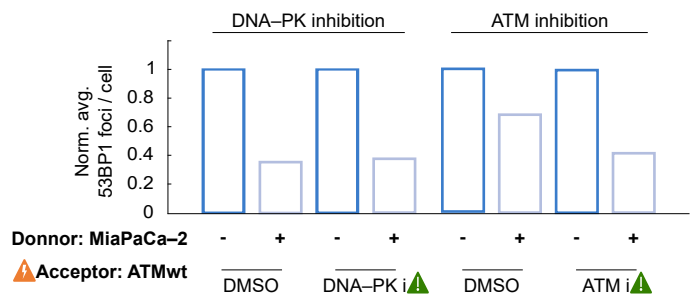

Supplement: Supplementary file 10 — Additional file 10: Figure S10. Combination of DNA–PK or ATM inhibitor treatment and irradiation in mono– and co–culture. DNA–PK or ATM inhibitor treated, 6–Gy irradiated ATMwt fibroblasts (acceptor) cultured alone or in co–culture with untreated MiaPaCa–2 cells (donor). Residual, 24–h 53BP1 foci in irradiated ATMwt acceptor cells are analysed. Relative 53BP1 foci values are displayed, corresponding to Fig. 3a. [file 12964_2019_472_MOESM10_ESM.pdf]

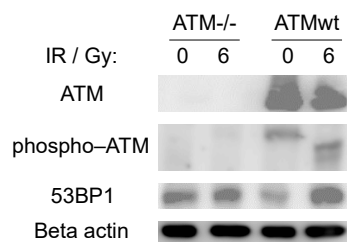

Supplement: Supplementary file 11 — Additional file 11: Figure S11. Expression of ATM, phospho–ATM S1981 and 53BP1 in ATMwt and ATM−/− fibroblasts. Western blot of protein expression in whole cell lysates from 6–Gy x–ray irradiated or unirradiated ATMwt and ATM−/− fibroblasts after 24 h. Beta actin was used as loading control. [file 12964_2019_472_MOESM11_ESM.pdf]

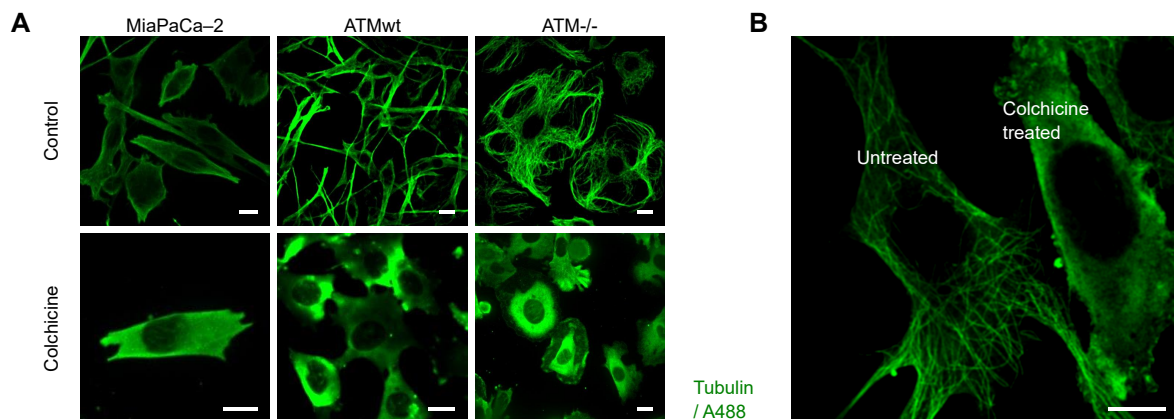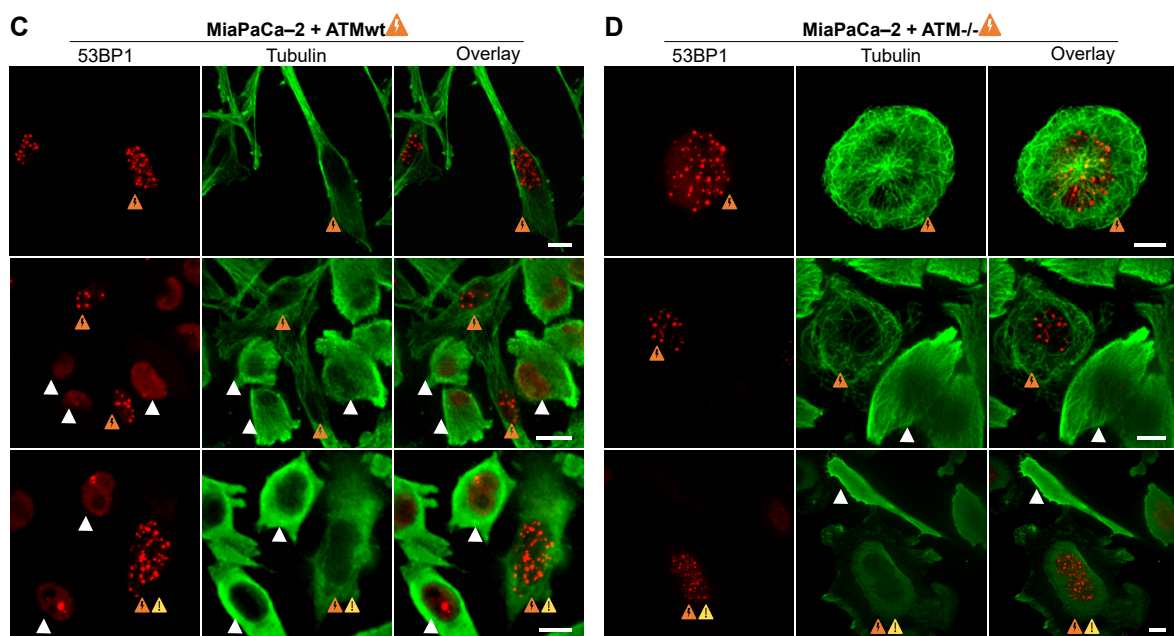

Supplement: Supplementary file 12 — Additional file 12: Figure S12. DSB repair depends on microtubule networks. a Representative images showing immunofluorescence staining of microtubules in MiaPaCa–2 cells, ATMwt and ATM−/− fibroblasts treated with 30 μM colchicine or left untreated. b Representative high–resolution image of untreated and colchicine treated cells in co–culture. c–d Corresponding to Fig. 4a. Irradiated ATMwt and ATM−/− fibroblasts with or without colchicine pretreatment were either plated for mono– or co–culture together with untreated MiaPaCa–2 cells (white triangles). Representative maximum intensity projections of z–stacks visualizing 53BP1 foci (anti–53BP1 / Alexa594) in ATMwt or ATM−/− fibroblasts and microtubules (anti–tubulin / A405) using immunofluorescence staining. Scale bars, 10 μm. [file 12964_2019_472_MOESM12_ESM.pdf]
